# Responsible handling of ethics in data publication

**DOI:** 10.1371/journal.pbio.3001606

**Published:** 2022-03-28

**Authors:** Daniella Lowenberg, Iratxe Puebla

**Affiliations:** 1 California Digital Library, University of California Office of the President, Oakland, California, United States of America; 2 ASAPbio, Cambridge, United Kingdom

## Abstract

A global working group has developed recommendations for the responsible handling of research data publication ethics, but there are further steps that repositories, publishers, institutions, and researchers can take in support of responsible open data publishing.

## Teaser

A global working group has developed recommendations for the responsible handling of the growing range of ethics cases related to data publication, but further community work is needed toward a responsible and cohesive data publishing ecosystem.

Integrity is a necessary driver of research quality and is foundational for developing trust in research. Research integrity has been at the forefront of discussions among funders, publishers, and the research community, who recognize the need for culture change that addresses perverse research incentives and challenges around diversity and inclusion [1–3]. In the context of journal publications, editors and publishers have developed best practices to address ethical and research integrity issues (such as those from the Committee on Publication Ethics (COPE) or the International Committee of Medical Journal Editors (ICMJE)) that have been widely adopted across scientific journals.

Beyond the journal article, data sharing has been fueled by funder mandates, institutional support, data policies at journals [4,5], and the growth in the number of repositories [6]. Initiatives for data citation and data assessment metrics are driving recognition of datasets as research outputs in their own right. However, this rise in the number of public datasets has led to comparable growth in the number and range of ethical issues associated with datasets.

For datasets to be recognized as important research contributions, researchers must have a rigorous publishing environment that provides safeguards, should concerns arise. In turn, researchers reusing the data need reassurance that the dataset is in good standing. Therefore, institutions, data repositories, and journals must have standards that create a safe space for researchers and allow them to address concerns consistently and responsibly. Such standards exist for journal articles but have not historically been in place for data, and repositories had not been involved in conversations related to ethics in data publication.

To address this gap, we created the FORCE11 Research Data Publishing Working Group, in collaboration with COPE. This collaboration leverages FORCE11’s expertise with data-related initiatives for scholarly communication and COPE’s expertise in developing guidelines for editors and publishers. The Working Group brought together researchers, data repositories, journals, and institutions (research integrity officers and libraries). The group has developed 4 categories for ethics concerns—Authorship & Contribution Conflicts, Legal & Regulatory Restrictions, Rigor, and Risk—and practical guidance for follow up on cases in each category, according to the publication status of the dataset (not yet published or published). The recommendations also cover when to report concerns to institutions and handling communications between the data publisher and other parties. We shared the recommendations [7] in September 2021 and invited the community to incorporate them into their practices and share feedback on needed refinement or further development.

While the recommendations reflect input from a diverse group, the discussions within the Working Group highlighted several areas of data publication that require further community discussion (Box 1).

**Box 1. Areas of data ethics that require further community discussion.**

- **Resources available at repositories:** Among the thousands of data repositories (institutionally based, generalist (commercial or open source), or discipline specific), resourcing for data curation and author help desk ranges drastically. The community needs to further explore how to reconcile broad recommendations with what is practical for implementation.
- **Legal cases:** Legal frameworks vary by country or even by state, and it is not possible to build blanket recommendations for cases that involve legal considerations. Journals and repositories do not all have legal counsel accessible. The tension between the urgency to respond to a legal challenge and the often lengthy timeline when a case is escalated to an institution needs to be addressed.
- **Terminology:** Posting notifications on datasets is a new and developing practice for data repositories that will require consensus for language. Whether tags such as “retraction” and “expression of concern” are appropriate for data records requires further discussion.

In parallel to these open questions, Findable, Accessible, Interoperable, and Reusable (FAIR) data publishing must adapt to support ethical best practices for open data. We propose several steps that can be taken to build a more cohesive and responsible data publishing network.

First, the community should value data permanency and quality curation. This includes encouraging researchers to deposit data in repositories that assign a persistent identifier-based citation (e.g., a DOI) and incorporate robust curation prior to publication. If the community aims to collectively value data as a first-class object, data must be published only within frameworks that ensure that data are complete, reusable, and understandable. For example, Dryad completes curation of submitted datasets for completeness of data files and accuracy of metadata prior to publishing the dataset.

Second, peer review of data needs to be built up. Peer review of data as common practice will enable any concerns to be identified earlier and more effectively before publication. Communicating any such concerns to the repository allows the data to be changed or removed before being broadly accessible online. We understand that peer review is already a time-consuming process, but because the findings in the article rely on the data, it must be part of the review process. Some journals have introduced specialized review steps [8], and there are also initiatives to support the review of code [9]. *The Lancet* recently implemented specific requirements for papers involving large datasets [10] including that for those submissions, at least 1 reviewer should have expertise in the dataset reported. We should explore new approaches to the review of data, beginning with leveraging “private for peer review” features at repositories. If data are published without a related article, we should find ways to incorporate community peer review into repository workflows; different groups are experimenting with the review of code and preprints to provide public feedback on those outputs; similar approaches could be explored for datasets. In addition, we should empower repositories to post a notification on the dataset when integrity concerns arise.

Third, repositories and publishers need to establish open and definitive lines of communication around research integrity issues. Data statements inform journals about where the data are hosted, but few repositories have information on where a related manuscript is under review (or published) or have the relationships with publishers through which to easily communicate about concerns that arise. There is a need for additional forums within multistakeholder organizations to allow such cross-communication between journals and data repositories.

Finally, we need further researcher education, both preventatively and during the handling of individual cases. For example, data files are often published that should not be licensed with a CC0 waiver of license. Researchers must learn about what it means to publish data. To do so, it will be important to support researcher education even before the research begins (e.g., at the data management plan stage or through instruction of responsible conduct of research).

Open and FAIR data practices must keep integrity and ethics at their core to build the trust that researchers need to responsibly share, access, and reuse datasets. The research community must collectively establish ethical practices in data publication, accounting for the needs of various stakeholders and establishing educational measures that support researchers throughout the data publication process. We look forward to building a community of support around the responsible handling of ethical considerations in research data publication.

## Abbreviations

COPE: Committee on Publication Ethics
FAIR: Findable, Accessible, Interoperable, and Reusable
ICMJE: International Committee of Medical Journal Editors
